# Unusual Presentation of *Mycobacterium abscessus* Thigh Mass: A Case Report

**DOI:** 10.1155/crdi/6970929

**Published:** 2025-07-09

**Authors:** Ryan J. Blake, Grant R. McChesney, H. James Williams, Steven M. Holland, Allison M. Lastinger

**Affiliations:** ^1^Department of Orthopaedic Surgery, West Virginia University, Morgantown, West Virginia, USA; ^2^Musculoskeletal Infection Program, West Virginia University, Morgantown, West Virginia, USA; ^3^Department of Pathology, Anatomy, and Laboratory Medicine, Morgantown, West Virginia, USA; ^4^National Institute of Allergy and Infectious Disease, National Institute of Health, Bethesda, Maryland, USA; ^5^Department of Medicine, West Virginia University, Morgantown, West Virginia, USA

**Keywords:** abscess, *Mycobacteria*, resistance, skin and soft tissue

## Abstract

*Mycobacterium abscessus* is a rapidly growing non-*tuberculous Mycobacterium* (NTM) primarily associated with pulmonary infections, particularly in individuals with underlying lung conditions. While soft tissue infections are less common, their incidence has been increasing. These infections are challenging to treat due to inherent resistance to many antibiotics obtained through spontaneous mutation as well as physical characteristics of the microbes. The case presented here describes a 61-year-old female without obvious risk factors for mycobacterial infection who developed an intramuscular abscess over a 2-year period following a mechanical fall. Surgical resection with a complex antibiotic regimen was required based on macrolide resistance and a lack of established treatment plans for such a rare presentation. This case highlights the increasing incidence of NTM and the variable clinical presentation. Early identification with a combination of surgery and antibiotics is usually indicated to successfully manage these infections. Even without obvious risk factors, NTM infection should be considered in the presence of soft tissue and intramuscular abscesses.

## 1. Introduction

The genus *Mycobacterium* is a large collection of bacterial organisms characterized primarily by their molecular sequence coding for the 16S rRNA, a component of the prokaryotic 30S ribosomal subunit [1]. The organisms are gram-positive, acid-fast rods [1]. Other bacteria such as *Corynebacterium* and *Nocardia* have similar molecular structures compared to that of *Mycobacterium* based on a large composition of guanine and cysteine, which all combine to create the subgroup Actinobacteria [1, 2]. *Mycobacterium* is specifically known for its relatively slower growth rate compared to others in the Actinobacteria class and opportunistic pathogenicity affecting pulmonary systems [1, 3, 4]. The genus can be further divided into two groups: *Mycobacterium tuberculosis* and non-*tuberculosis Mycobacteria* (NTM), the latter of which includes *M. abscessus*, *M. bolletii*, and *M. massiliense*. *M. abscessus* is native to natural, moist environments and has frequently been described as opportunistic, affecting those who are immunocompromised. However, the increasing incidence of this *Mycobacteria* regardless of immune status suggests an evolution from the opportunistic pathogen to an organism capable of increasing virulence [3].

*M. abscessus* is the second highest cause of NTM infection of the lung after *Mycobacterium avium* complex [5]. The organism most commonly affects those with underlying lung disease or those with diminished immune responses. Cystic fibrosis, chronic obstructive pulmonary disease, bronchiectasis, and prior tuberculosis are such pathologies with predisposing risk for acquiring an infection with *M. abscessus* [6], and some sources quote a one-thousand-fold increase in risk associated with a diagnosis of cystic fibrosis, specifically [3]. Presentation of *M. abscessus* is variable and can follow that of many conditions affecting the lung, but common findings are chronic or recurring cough with constitutional symptoms [6]. Diagnosing infection with the *Mycobacteria* depends on established guidelines characterized by positive cultures from sputum, positive cultures from bronchial lavage, or lung biopsy with granulomatous tissue inflammation combined with a positive culture from either sputum or bronchial lavage [6].

Following colonization of the lung, the skin and soft tissue are the second most common primary sites of infection with *M. abscessus*, a NTM [7]. The causative route for infection usually involves contact of damaged skin with contaminated soil or water where the organisms reside, but cutaneous lesions lack specificity in identifying specific organisms. There are some reports of patients developing NTM infection after medical intervention, as well [4, 7]. Intramuscular, tendon, and bone infections secondary to *M. abscessus* are exceedingly rare.

Infection with *M. abscessus* and other non-*tuberculosis* species remains difficult to treat with antibiotics due to intrinsic characteristics conferring resistance to most modern antibiotics [3]. In addition, there remains a lack of consensus on treatment strategies due to the general rarity of infection, specifically 1.3 cutaneous infections per 100,000 person-years [8]. Treatment regimens for these cutaneous infections are limited to sparse reports in the literature [4], but most are treated with a macrolide in combination with amikacin and surgical debridement since antibiotic treatment alone is usually not sufficient to overcome the pathogen [9]. Given the rarity of muscular infection secondary to this organism, optimal treatment is not well-defined. Here, we present the unique case of a patient who developed an intramuscular abscess in their left leg over a 2-year period after sustaining a fall that eventually cultured positive for *M. abscessus* and required surgical resection with antibiotic treatment. The patient provided written consent for this publication.

## 2. Case

**Figure 1:**
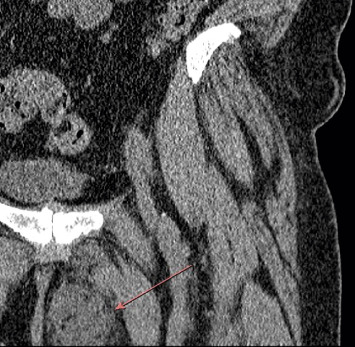
CT scan demonstrating a marginated cystic mass in the subcutaneous perineal fat.

A 61-year-old female presented to our musculoskeletal infectious disease department with concerns for an abscess in the left thigh. Approximately two and a half years prior, the patient suffered a mechanical fall after slipping on ice. Following the fall, a mass developed in the left groin that continued to grow over the next few months with eventual spontaneous rupture. Continuous draining from the groin prompted the patient to seek medical care at their local emergency department, where a computed tomography (CT) imaging study was performed that revealed a possible perineal hematoma (Figure 1). Cultures of the wound were obtained in the emergency department and were positive for coagulase-negative staphylococci. The patient was discharged on sulfamethoxazole–trimethoprim and referred to a general surgeon.

The patient reported that it felt as though the abscess had collected fluid in the few days between the emergency department visit and the appointment with the general surgeon. Induration, cellulitis, and purulent drainage were noted on physical examination of the perineum by the surgeon, so an incision and drainage of the abscess were decided upon. Unfortunately, the abscess continued to collect purulent material over the following weeks, and the patient was able to express purulent material on a few occasions. However, there was no further drainage of the abscess.

**Figure 2:**
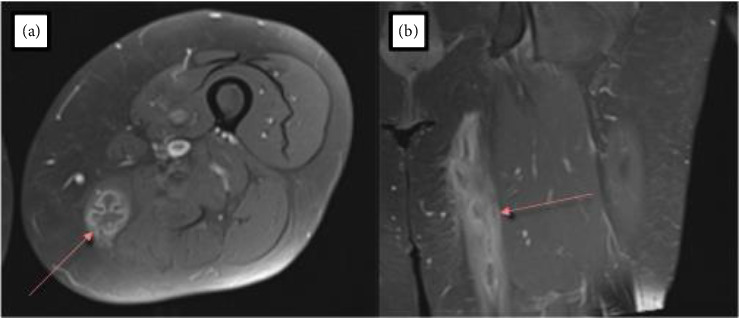
(a) Axial postcontrast T1-weighted MRI demonstrating abscess formation in the gracilis and (b) coronal view showing longitudinal gracilis involvement.

Approximately 15 months after the initial fall on the ice, the mass in the perineum/thigh region began to increase in size and cause significant irritation. The patient reported that the left groin abscess never truly disappeared following initial treatment. Ultrasound examination of the thigh at this time further characterized the palpable mass as a 3.4 × 7.9 × 11.0 cm possible hematoma with solid character. Due to the latter, the patient was referred to an orthopedic oncologist at our institution for additional evaluation. An MRI of the thigh demonstrated a mass centered along the intramuscular tendon of the left gracilis muscle with extension into the posterior medial subcutaneous tissues of the thigh along with a small intramuscular hematoma in the vastus lateralis muscle (Figure 2). An interventional radiology aspiration of the mass yielded a positive culture for *M. abscessus,* subspecies *abscessus*. The organism was also sent to a reference lab where it was found to have resistance to macrolides but susceptibility to aminoglycosides (Table 1). An infectious disease specialist at our institution subsequently started the patient on amikacin, eravacycline, and linezolid while also consulting the National Jewish *Mycobacterium* Consult Service. Imipenem was additionally added to the regimen.

**Figure 3:**
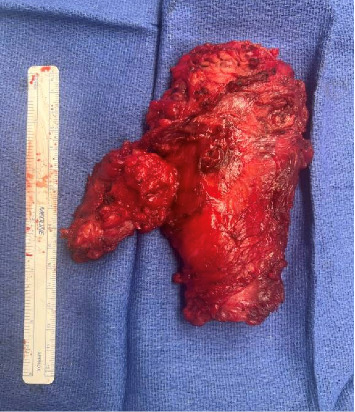
Resection specimen (10.5 × 8.0 × 4.1 cm) comprised of the gracilis muscle and resected sinus tract.

**Figure 4:**
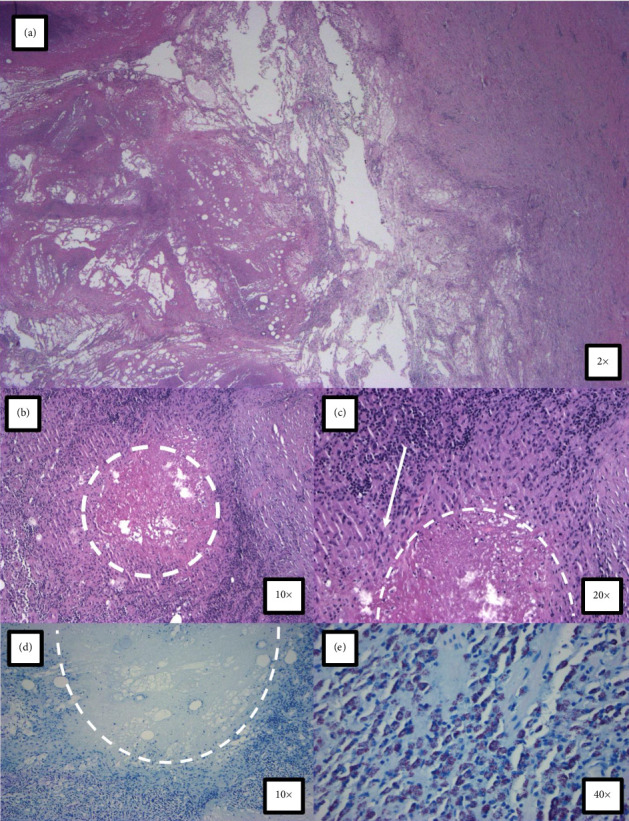
(a) Low magnification demonstrating extensive necroinflammatory debris consistent with abscess, H&E 2x; (b) rare caseating granuloma seen in association with the abscess (white circle), H&E 10x; (c) high magnification showing palisading histiocytes (white arrow) around central necrosis (white hemicircle), H&E 20x; (d) caseating granuloma (white hemicircle) in patient specimen negative for acid-fast bacillus (AFB) stain, AFB 10x; (e) AFB Kinyoun controls slide stains appropriately, AFB 40x.

Due to the high likelihood of recurrence with antibiotics alone, a surgical resection was deemed necessary to treat the infection with the NTM. Intraoperatively, the mass was found to occupy the middle portion of the gracilis muscle with most of the posterior belly involved. The muscle was grossly infected, and the abscess was removed with the entirety of the mass. The abscess significantly compromised the gracilis muscle, and a sinus tract located near the origin of the proximal gracilis muscle was also removed. The muscle was widely excised around the abscess for tissue margin (Figure 3). Operating room cultures were positive for methicillin-sensitive *Staphylococcus aureus*, *Corynebacterium aurimucosum*, and *Mycobacterium abscessus* subspecies *abscessus*. Surgical pathology and microscopic examination were consistent with necrotizing granulomatous inflammation with an acute abscess formation but negative for special stains for acid-fast bacilli and fungi (Figure 4). There was an absence of dysplastic growth or evidence of neoplasm. The patient was discharged with plans for long-term antibiotic therapy of approximately 6–12 months.

## 3. Discussion

Infection with *M. abscessus* is one of the main contributors to nontuberculosis lung disease. The organism is also known to colonize skin and soft tissue leading to infection and abscess formation, but the incidence is generally lower than that of lung disease [10]. However, the relative rate of soft tissue involvement has been steadily increasing over the previous 2 decades [8]. This case contributes to other reports describing cutaneous manifestations of NTM infection, but this case is particularly unique given the intramuscular nature of the abscess which is uncommon for *M. abscessus*. This case is unique because the patient did not have apparent risk factors other than diabetes mellitus Type II [10], which was abnormal (hemoglobin A1C = 9.6%) 1 month prior to surgical resection. There were no apparent medical interventions consistent with contamination of *M. abscessus* or any obvious contact with soil or water housing the microbe. It is important to note that incisions and drainage of the abscess were performed on multiple occasions over an approximate 2-year period, but these procedures were in response to the initial formation of the mass and did not occur before initial symptoms began, lessening the likelihood that these incisions induced the infection. A CT of the chest did not suggest that the organism was acquired from the lungs and then disseminated to the left thigh.

The *M. abscessus* organism in this case was resistant to macrolides, and initial treatment strategies are dependent on the presence of this resistance since macrolides represent one of the few classes that is able to treat the infection. Genotype and phenotype analyses of *M. abscessus* strains have highlighted multiple mechanisms responsible for the decreased susceptibility to most antibiotic classes. These mechanisms include drug export systems in the forms of efflux pumps, presence of enzymes capable or altering drugs, polymorphisms in target genes, and an impermeable cell wall acting as a size and chemical filter [11]. Macrolides are most commonly paired with a tetracycline, amikacin, cefoxitin, or clofazimine, when the organism is susceptible to minimize antibiotic resistance [12]. In our patient's case, macrolide resistance was predicted; therefore, she was given an aminoglycoside (amikacin 15 mg/kg 3 times weekly), a tetracycline (eravacycline 1 mg/kg IV every 12 h), and an oxazolidinone (linezolid 600 mg daily) consistent with most treatment reports in the literature. While on the initial regimen, the patient developed vestibular toxicity from the aminoglycoside and severe nausea and vomiting from eravacycline; thus, her regimen was adjusted to omadacycline 300 mg daily, linezolid 600 mg daily, and combination meropenem plus ceftaroline (administered as meropenem 1 g IV every 12 h followed by ceftaroline 600 mg IV every 12 h).

NTM can have intrinsic and acquired forms of resistance against antimicrobial therapies. The intrinsic mechanisms involve the physical structure of the pathogen, in which *Mycobacteria* contain a thicker cell wall that is generally impermeable with a slightly altered biochemical composition. The peptidoglycan layer of these microbes contains an N-glycol muramic acid instead of the N-acetyl muramic acid, and long-chain fatty acids with upward of 90 carbons contribute to a hydrophobic environment not suitable for most drug therapies [13]. These characteristics increase the likelihood of forming biofilms and granulomas as the body attempts to contain or eliminate the infection, accounting for the necrotizing granulomatous tissue seen in the surgical resection of the abscess. Acquired forms of resistance have demonstrated multiple different mechanisms. However, macrolide-specific resistance has been reported to involve mutations in *erm* genes responsible for methylating the 23S ribosomal RNA, rendering macrolides ineffective [13]. The relative lack of ribosomal RNA operons in NTM species means that single mutations can substantially alter the structure of the ribosome. Results of genotyping from the National Jewish *Mycobacterium* Consult Service confirmed a mutation in the *erm* (41) gene for this patient. The resistance in the strain presented here could have provided the protection that allowed the abscess to form over a longer period of time.

Treatment of this patient was unique because abscess formation occurred over a 2-year time frame where initial presentation was prompted by a fall. Most cases of *M. abscessus* involve colonization of the lung, and the cutaneous forms of the infection result from contact with contaminated instruments, especially during medical intervention. Therefore, the temporal characteristics of the abscess combined with the clinical context of the patient increased the likelihood of a neoplasm compared to that of bacterial infection, but a positive aspiration yielded *M. abscessus*, an uncommon pathogen. Due to the intrinsic resistance of the non-tuberculosis organism, surgical intervention in addition to a complex antibiotic regimen was deemed necessary to effectively manage the infection. This case highlights the need for increased clinical suspicion of *Mycobacteria* infection in the context of intramuscular abscess formation even without obvious risk factors since there is an increasing incidence of NTM infection.

## Figures and Tables

**Table 1:** Antibiotic susceptibility following aspiration of the abscess.

Antibiotic	Minimum inhibitory concentration (μg/mL)	Level of susceptibility
Amikacin	≤ 8	Susceptible
Augmentin	> 32/16	NI
Azithromycin	≤ 16	NI
Azithromycin (14 day)	> 256	NI
Cefepime	> 32	NI
Cefotaxime	64	NI
Cefoxitin	≤ 16	Susceptible
Ceftriaxone	> 64	NI
Ciprofloxacin	2	Intermediate
Clarithromycin	≤ 0.25	Susceptible
Clarithromycin (14 day)	> 32	Resistant
Clofazimine	≤ 0.5	NI
Clofazimine/amikacin	≤ 0.5/2	NI
Doxycycline	> 16	Resistant
Gentamycin	8	NI
Imipenem	≤ 2	Susceptible
Kanamycin	≤ 8	NI
Linezolid	8	Susceptible
Minocycline	8	NI
Moxifloxacin	2	Intermediate
Tigecycline	≤ 0.25	NI
Tobramycin	8	Resistant
Trimethoprim/sulfamethoxazole	4/76	Resistant

*Note:* NI: No interpretive guidelines for this antibiotic/organism combination.

## Data Availability

All data generated or analyzed during this study are included in this article.

## Supporting Information

According to the CARE guidelines (for CAse REports), a checklist was completed for this manuscript, which can be found as supporting information.
